# Baseline Single‐Cell Differences in Polyfunctionality Between Systemic Autoinflammatory Diseases Patients and Healthy Controls

**DOI:** 10.1002/eji.70047

**Published:** 2025-09-03

**Authors:** Aline Linder, Farah Diab, Loïc de Pontual, Irina Giurgea, Klaus Eyer

**Affiliations:** ^1^ Laboratory For Functional Immune Repertoire Analysis, Department of Chemistry and Applied Biosciences ETH Zurich Zurich Switzerland; ^2^ Sorbonne Université, Inserm Maladies génétiques d'expression pédiatrique Paris France; ^3^ Service de Pédiatrie Hôpital Jean Verdier Bondy France; ^4^ Sorbonne Université, APHP Hôpital Armand‐Trousseau Paris France; ^5^ Department of Biomedicine Aarhus University Aarhus Denmark

## Abstract

Dysregulated cytokine secretion and signaling underlie systemic autoinflammatory diseases (SAIDs). Here, we characterized immune dysregulation in SAID patients by profiling cytokine secretion at the single‐cell level, establishing measurements for secretion dynamics and cellular polyfunctionality, compared with healthy controls, revealing natural variability within immune responses between donors.

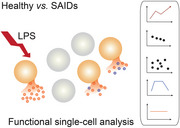

If not properly regulated, activation of inflammatory pathways can cause immune‐mediated disorders such as systemic autoinflammatory diseases (SAIDs) [1, 2]. SAIDs are rare, heterogeneous conditions that include both monogenic and multifactorial forms [1, 2]. They typically are due to mutations in components of the innate immune system [3], leading to recurrent inflammatory episodes [4]. A well‐described example of SAID is tumor necrosis factor receptor‐associated periodic syndrome (TRAPS) [5]. However, defects in various other pathways can also underlie SAIDs, complicating both the diagnosis and treatment [1, 2], as disease‐causing mutations are usually found in only 40%–60% of cases [4, 6]. Rather than relying on often‐normal plasma levels during active disease [7], we utilized a single‐cell functional analysis platform capable of quantifying cytokine secretion from individual immune cells [8, 9]. This method captures cytokine signatures in detail and reveals differences in polyfunctionality (Figure 1) [10], offering high‐resolution insights into immune dysregulation (Methods, Figure S1).

**FIGURE 1:**
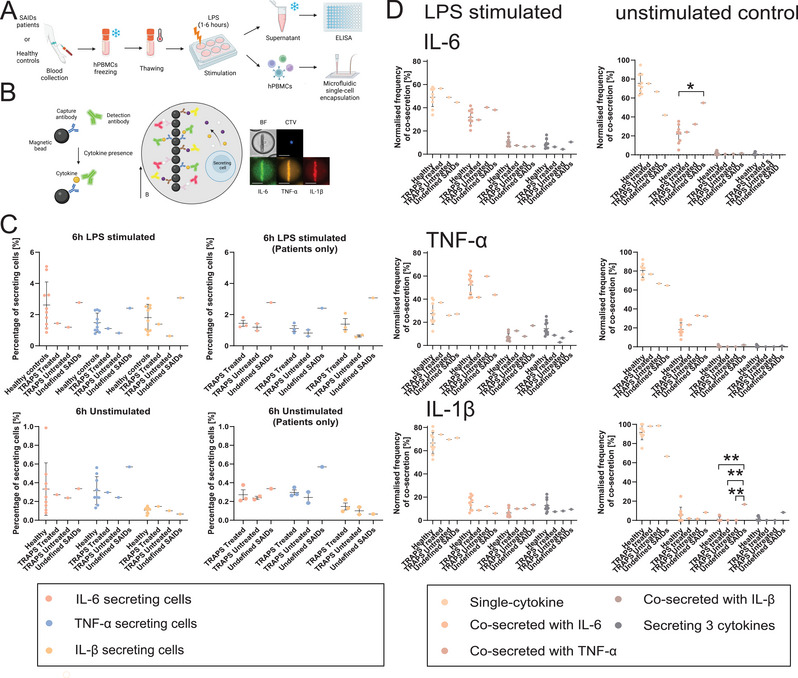
(A) Overview of the workflow. (B) A sandwich immunoassay using magnetic capture beads and fluorescent detection antibodies quantified IL‐6, TNF‐α, and IL‐1β secretion from individual cells. Fluorescence relocation indicated secretion, as shown in the micrograph for a cell secreting all three cytokines. Created with BioRender.com, scale bar: 25 µm. (C) Frequencies of cells secreting IL‐6, TNF‐α, and IL‐1β after 6 h LPS stimulation or unstimulated, and (D), the same data resolved for polyfunctionality. From left to right: co‐secretion with IL‐6 as a base, followed by TNF‐α and IL‐1β. Data shown as mean ±SD for healthy controls (*N *= 10) and mean ±SEM for patient replicates (TRAPS_Treated_ *N *= 1/*n *= 3; TRAPS_Untreated_ *N *= 1/*n *= 2; SAID_Undefined_ *N *= 1/*n *= 1). One‐way ANOVA with Tukey's post hoc test; *p < 0.05, **p < 0.01.

We first assessed the frequency of cytokine‐secreting cells (SCs) producing IL‐6, TNF‐α, and IL‐1β in healthy controls and SAIDs patients. Looking at the basal immune response after 6 h incubation (healthy donors, *n* = 10, without stimulation), we found 0.33 ± 0.28% IL‐6‐SCs, 0.32 ± 0.15% TNF‐α‐SCs, and 0.11 ± 0.03% IL‐1β‐SCs (Figure 1C). Upon stimulation with LPS, the healthy control group displayed a 5‐to‐16‐fold increase. A large variation in the frequencies of SCs was notable within the healthy individuals, highlighting a large variability in the proportion of activated cells in healthy individuals in response to LPS.

Our small exploration cohort comprised SAIDs patients, including two individuals diagnosed with TRAPS and a patient with undefined SAID (Table S1; Figure 1C). Regarding both TRAPS samples, they displayed similar frequencies to healthy controls of SCs after stimulation with LPS for 6 h (IL‐6‐SCs 1.44 ± 0.19% and 1.19 ± 0.23%, TNF‐α 1.10 ± 0.19% and 0.81 ± 0.20%, and IL‐1β 1.39 ± 0.36% and 0.63 ± 0.07%, respectively). This similarity was also observed in unstimulated conditions. These experiments showed that replicate measurements of the same sample varied less than those between healthy donors, suggesting that donor variation reflects biological differences rather than measurement error. The undefined SAID sample (measured once due to low availability), was nonsignificantly different in cytokine‐SCs frequencies compared with healthy controls in both conditions.

Second, it was further possible to identify the co‐secreting behavior of various subpopulations (Figure 1D). Here, IL‐6+TNF‐α refers to IL‐6 SCs that also co‐secrete TNF‐α, whereas TNF‐α+IL‐6 would consequently refer to TNF‐α SCs that also co‐secrete IL‐6. In healthy donors and after 6 h of stimulation with LPS, IL‐6 was mostly secreted by itself (49.1 ± 8.1%) or co‐secreted with +TNF‐α (31.7 ± 6.6%), with little co‐secretion with +IL‐1β (10.5 ± 3.7%) or with both (+IL‐1β+TNF‐α 8.6 ± 3.9%). TNF‐α, on the other hand, was in the majority co‐secreted with +IL‐6 (52.2 ± 8.5%), and to a lesser extent, secreted alone (27.5 ± 9.3%) or in combination with +IL‐1β (6.9 ± 3.1%) or with +IL‐6+IL‐1β (14.6 ± 5.7%). Lastly, IL‐1β was primarily secreted by itself (66.5 ± 9.1%), to a lesser degree co‐secreted with +IL‐6 (15.3 ± 5.6%), +TNF‐α (6.5 ± 3.9%) or both (12.7 ± 5.2%). Without stimulation, the observed polyfunctionalities were largely decreased in healthy donors, as expected, as the three cytokines were primarily secreted by themselves.

Interestingly, already unstimulated PBMCs of the undefined SAID patient showed an increase in co‐secretion with +TNF‐α, of IL‐6 and IL‐1β SCs. In this sample, IL‐6 was secreted by itself in only 41.9% of all IL‐6‐SCs (compared with 75.5 ± 10.7% in healthy donors, *p* = 0.07). The co‐secretion with +TNF‐α corresponded to 54.8% of all IL‐6‐SCs, which was a clear increase compared with healthy controls (21.9 ± 9.6%, *p* = 0.046). Additionally, IL‐1β single‐secretion was observed in only to 66% of all cells secreting IL‐1β (91.7 ± 7.7% in healthy controls, *p* = 0.059), and the co‐secretion with +TNF‐α was increased to 16% compared with the control group (1.2 ± 2.3%, *p* = 0.001), and TRAPS samples (0 ± 0% in both cases, *p* = 0.0043). Interestingly, these differences disappeared after stimulation (Figure 1D, first row). Hence, in the absence of stimulus, the immune cells of the undefined SAID patient displayed an increase in the prevalence of co‐secreted TNF‐α compared with the other groups.

Next, we further focused on the secreted amounts and rates, focusing on the 6 h time point. Secreted concentrations and secretion rates (SRs) varied with polyfunctionality in LPS‐stimulated cells but were similar between healthy and SAIDs patients (Figure 2). Specifically, the secretion of IL‐6 displayed differences according to the co‐secretion groups (Figure 2A). When IL‐6 was co‐secreted with +TNF‐α or with +TNF‐α+IL‐1β, the maximum secreted concentration of IL‐6 was higher compared with the other groups (IL‐6 alone, or +IL‐1β, *p* < 0.0001). These higher concentrations were due to increased SRs (Figure 2B), where cells secreting IL‐6+TNF‐α or IL‐6+TNF‐α+IL‐1β had a 5× higher median IL‐6 SR (*p* < 0.0001). TNF‐α secretion was similar across co‐secreting subpopulations. IL‐1β also displayed a differential secretion concentration and SR in +IL‐6 or +TNF‐α SCs. However, in this case, the maximal IL‐1β concentration as well as SR were increased in +IL‐6, +TNF‐α, and +IL‐6+TNF‐α SCs, compared with cells secreting only IL‐1β. Regarding the unstimulated conditions, the cells were mostly secreting single cytokines (Figure 2A). In summary, secreted concentrations and SRs at the single‐cell level showed no major differences between healthy and SAIDs patient samples, with all three patients falling largely within the normal range across categories.

**FIGURE 2:**
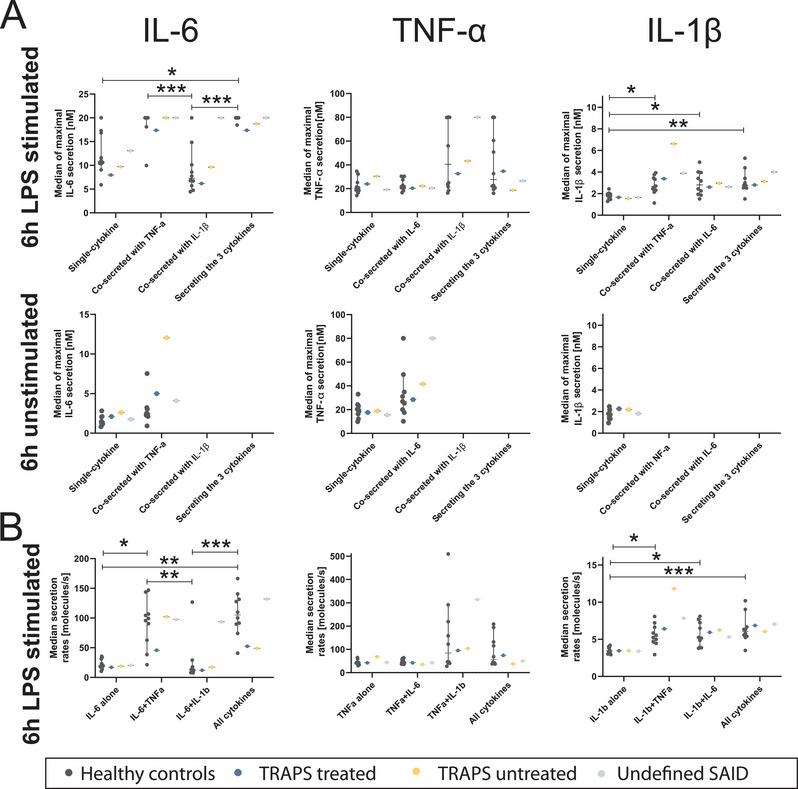
Median maximal secreted concentration (A) and SR (B) of IL‐6, TNF‐α, and IL‐1β after 6 h of LPS stimulation or unstimulated controls. Lines indicate median ± IQR (95%). Same samples as Figure 1, *n* < 5 SCs were excluded. Group differences were analyzed by Kruskal–Wallis with Dunn's post hoc test. Significance: *p < 0.05, **p < 0.01, ***p < 0.001.

We observed cytokine‐specific differences in the dynamics of secretion, which did not differ between patients and healthy individuals (Figure S5). Even when tested at a different time point, the secretion dynamics showed no significant differences between patients and controls (Figures S2–S4, S6). Bulk cytokine levels measured by ELISA confirmed these findings (Figure S7).

As a complement to genetic testing, we propose a method that directly measures cytokine secretion from individual immune cells, capturing both mono‐ and polyfunctionality alongside other cellular features. By layering functional data on top of genetic information, this method may help reveal the downstream effects of mutations and could be especially valuable for patients in whom no disease‐causing mutation is identified. In this study, we observed increased cytokine TNF‐α co‐secretion in resting immune cells from a patient with an undefined SAID, suggesting a distinct activation‐like phenotype not present in PBMCs from healthy controls or two TRAPS patients, nor reflected in serum measurements. Consequently, single‐cell cytokine profiling might reveal the consequences of mutations with unique immune signatures in SAIDs missed by genetic testing, but faces challenges from disease variability, potentially requiring adaptation. Therefore, different SAIDs may require tailored cytokine panels, making broad screening impractical, but might further enable personalized therapy after diagnosis thus, larger and more diverse studies are needed to evaluate their clinical value.

## Conflicts of Interest

The authors declare no conflicts of interest.

## Peer Review

The peer review history for this article is available at https://publons.com/publon/10.1002/eji.70047.

## Supporting information




**Supporting file 1**: eji70047‐sup‐0001‐SuppMat.pdf

## Data Availability

The data that support the findings of this study are available on request from the corresponding author.
