# Supplementary material for: Baseline Single‐Cell Differences in Polyfunctionality Between Systemic Autoinflammatory Diseases Patients and Healthy Controls
Source: Eur J Immunol. 2025 Sep 3;55(9):e70047. doi: 10.1002/eji.70047 (PMC12405973; doi:10.1002/eji.70047)
Supplement: Supplementary file 1 — Supporting file 1: eji70047‐sup‐0001‐SuppMat.pdf [file EJI-55-e70047-s001.pdf]

Supporting information

Baseline polyfunctional differences in single-cell phenotypes of SAIDs patients and healthy controls after high-dose LPS stimulation: A pilot study

Aline Linder[1], Farah Diab[2], Loic de Pontual[3], Irina Giurgea[2,4], Klaus Eyer[1,5#]

[1]Laboratory for Functional Immune Repertoire Analysis, Institute of Pharmaceutical Sciences, Department of Chemistry and Applied Biosciences, ETH Zürich, 8093 Zürich, Switzerland.

[2]Sorbonne Université, Inserm, Maladies génétiques d'expression pédiatrique, F-75012 Paris, France.

[3]Service de Pédiatrie, Hôpital Jean Verdier, F-93143 Bondy, France

[5]Sorbonne Université, APHP, Hôpital Armand-Trousseau, F-75012 Paris, France.

[5]Department of Biomedicine, Aarhus University, Bartholinbygningen, Wilhelm Meyers Allé 4, 8000 Aarhus C, Denmark.

# Correspondence: eyerk@biomed.au.dk

Methods

Participants. Individuals were included in this study after informed consent and consent to publish, which was approved by the ethics evaluation of the 'Institut national de la santé et de la recherche médicale' (INSERM, no: 21-851). The number of participants in each cohort alongside their sex, age and treatment at the time of sampling are shown in the **SI Table 1**.

hPBMCs isolation and freezing. In this study, blood from healthy donors and patients' samples were collected outside of flares, their hPBMCs were isolated, frozen and shipped for the functional assessment as described elsewhere[32]. There were some minor differences to the protocol described in Linder *et al.*, 800 g was used instead of 1000 g for centrifugations, and Pancoll (Pan Biotech) was used instead of Ficoll.

hPBMCs thawing, staining and stimulation. To thaw the cells , the cryogenic vials were swirled in a water bath at 37°C and the cells were immediately transferred into a centrifuge tube containing pre-warmed completed RPMI. After washing with completed RPMI, the cells were resuspended to a final concentration of $2x10^6$ cells/ml for staining with 5 μM CellTrace Violet (ThermoFisher) for 5 min at 37°C. After washing with MACS Buffer (PBS pH 7.2, BSA 0.5%, 2 mM EDTA, all SigmaAldrich), the cells were FcR-blocked using human FcR blocking reagent (Miltenyi Biotec) for 10 min at RT. After washing in completed RPMI, the cells were counted and resuspended in completed RPMI at a final concentration of 10^6 cells/ml. 2 ml of the cell suspension was seeded per well into an ultra-low binding 6-well plate (Corning), and the cells were either left unstimulated or stimulated with 1 μg/ml LPS (Invivogen) for 1 or 6 hours at 37°C. At the end of the stimulation, the supernatant was collected to be analyzed by ELISA, and the cells were thoroughly collected by scraping, washed and resuspended in completed RPMI at a concentration of $8x10^6$ cells/ml for encapsulation.

Microfluidic experiments and data analysis. The step-by-step protocol to perform the microfluidic experiment, as well as the assembly of the observation chamber, droplet generation and data analysis has been described in detail elsewhere[31, 32]. In short, we included sandwich immunoassays for three cytokines (TNF-α, IL-6, IL-1β) into each droplet. These assays consisted of nanoparticles that were modified with anti-cytokine antibodies to recognize the cytokine of interest, and the presence of cytokine was probed using a secondary, fluorescent-labelled anti-cytokine antibody. Calibration curves with recombinant proteins enabled quantification, with detection limits in low nanomolar to high picomolar ranges and maximum concentrations (see Figure S1 for calibration curves). The concentration of cytokine was detected via fluorescence relocation onto the nanoparticles, increasing the fluorescence on these particles and decreasing the in-droplet fluorescence consequently. This relocation value (intensity beads/intensity droplet) was calibrated with known cytokine concentrations (see Figure S1 for calibration curves). To analyze the unstimulated conditions, we only extracted the frequencies and maximal secreted concentration of cytokines, and the criteria for positive cells were limited to the limit of detection (LOD) only due to this fact (3 standard deviations). To calibrate the relocation signal into concentration, calibration curves using recombinant proteins have been used, and these can be found in Figure S1.

ELISA. Levels of IL-6, TNF-α and IL-1β in the supernatant were measured using ELISA kits (ThermoFisher) according to the supplier's instructions. Samples were run in duplicates, with 4 dilutions measured by 2-fold dilutions.

Statistical analysis. Analysis was performed using GraphPad Prism (version 9.3.1). Whenever specified, N corresponds to biological replicates (number of individuals) and n corresponds to technical replicates (number of replicates). For the frequencies, differences between the groups for each cytokine were assessed using a One-way ANOVA followed by Tukey's multiple comparisons. The different individuals are depicted with the mean ± standard deviation (SD), whereas the different measurements of an individual (i.e., repetition measurements of patients) are depicted on a separate graph with the mean ± standard error of the mean (SEM). For the concentration and secretion rate, the difference between the groups for each cytokine was assessed using a Kruskal-Wallis test, with a post-hoc Dunn's test. The differences between individuals and within runs of the same individual are depicted with the median ± IQR 95%. p-values were denoted as follows: * < 0.05, ** < 0.01, *** < 0.001, **** < 0.0001.

Author contributions. Aline Linder planned, performed, and analyzed the experiments and drafted the manuscript. Farah Diab and Loïc de Pontual collected and processed the healthy control and patient samples, Irina Giurgea and Klaus Eyer supervised the project. All authors commented on and revised the manuscript.

<u>Table S1</u>: Characteristics of the participants included in the study.

	Sex	Age (years)	Group	Treatment
Patient 1	F	37	TRAPS	Anakinra<br>(IL-1 agonist)
Patient 2	F	8	TRAPS	None
Patient 3	M	59	Undefined SAID	None
Healthy controls (n=10)	6 F<br>4 M	Median: 25.5<br>IQR: 24-35.25<br>Range: 22-61	Healthy controls cohort	None

Patient 2 is the daughter of patient 1. Both patients carry the heterozygous pathogenic variation c.236C>T, p.(Thr79Met) in the *TNFRSF1A* gene (NM_001065).

Patient 3 presented with adult-onset SAID, with no pathogenic variants identified in known SAID-associated genes tested through a targeted gene panel.

IQR: Interquartile range.

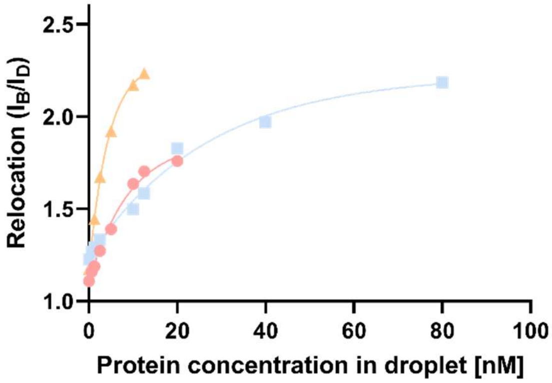

	IL-6	TNFα	IL-1β
Limit of detection	0.48nM	2.69nM	0.27nM
Maximum detection	20nM	80nM	12.5nM

<u>Figure S1</u>: Multiplexed cytokine calibration curves generated with known concentration of analytes and range of the assay. The relocation values and cytokines concentrations were fitted using a one-phase association curve fit with GraphPad Prism, with an R^2 of >0.99 for IL-6, TNF-α and IL-1β.

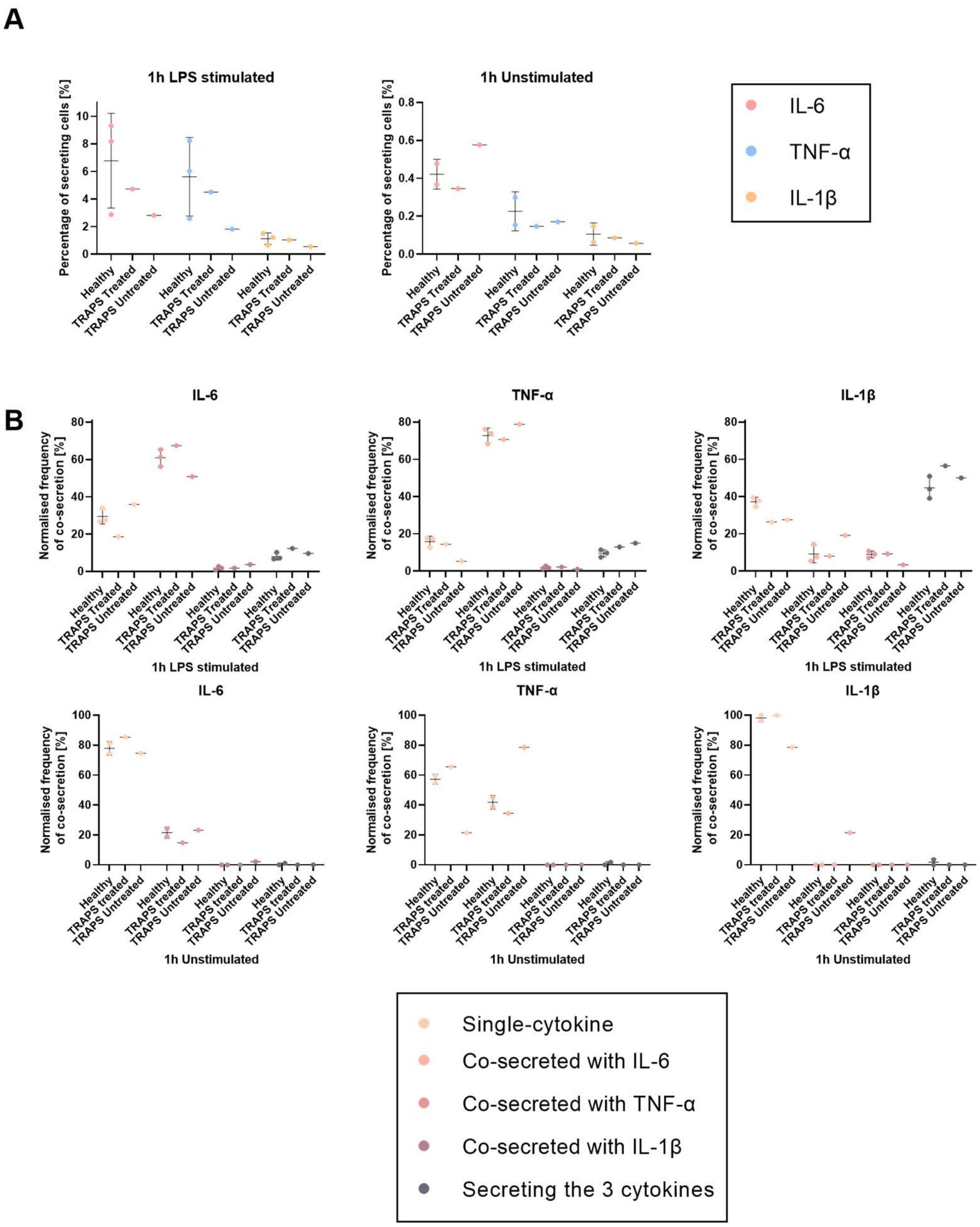

<u>Figure S2</u>: Frequencies of cytokines secreting cells after 1h stimulation. (A) General frequencies of cells secreting IL-6 (pink), TNF-α (blue) and IL-1β (yellow) cells after 1 h stimulation with LPS or unstimulated. Shown with average ±SD. No significant differences between the groups were found using a two-way repeated measures ANOVA. At 1-hour, healthy control group N=3, n=1; TRAPS Treated group N=1, n=1; TRAPS Untreated N=1, n=1. (B) Resolution of the polyfunctionality with the frequencies of co-secretion after 1 hour of stimulation with LPS or unstimulated. Depicted as average ±SD, with the single-cytokine secretion in black, co-secreted with IL-6 in orange, co-secreted with TNF-α in blue, co-secreted with IL-1β in pink and secreting the 3 cytokines in grey. Statistical differences were assessed with a one-way ANOVA followed by a Tukey's multiple comparison to compare between the co-secretion subgroups, no significant difference between the groups was observed.

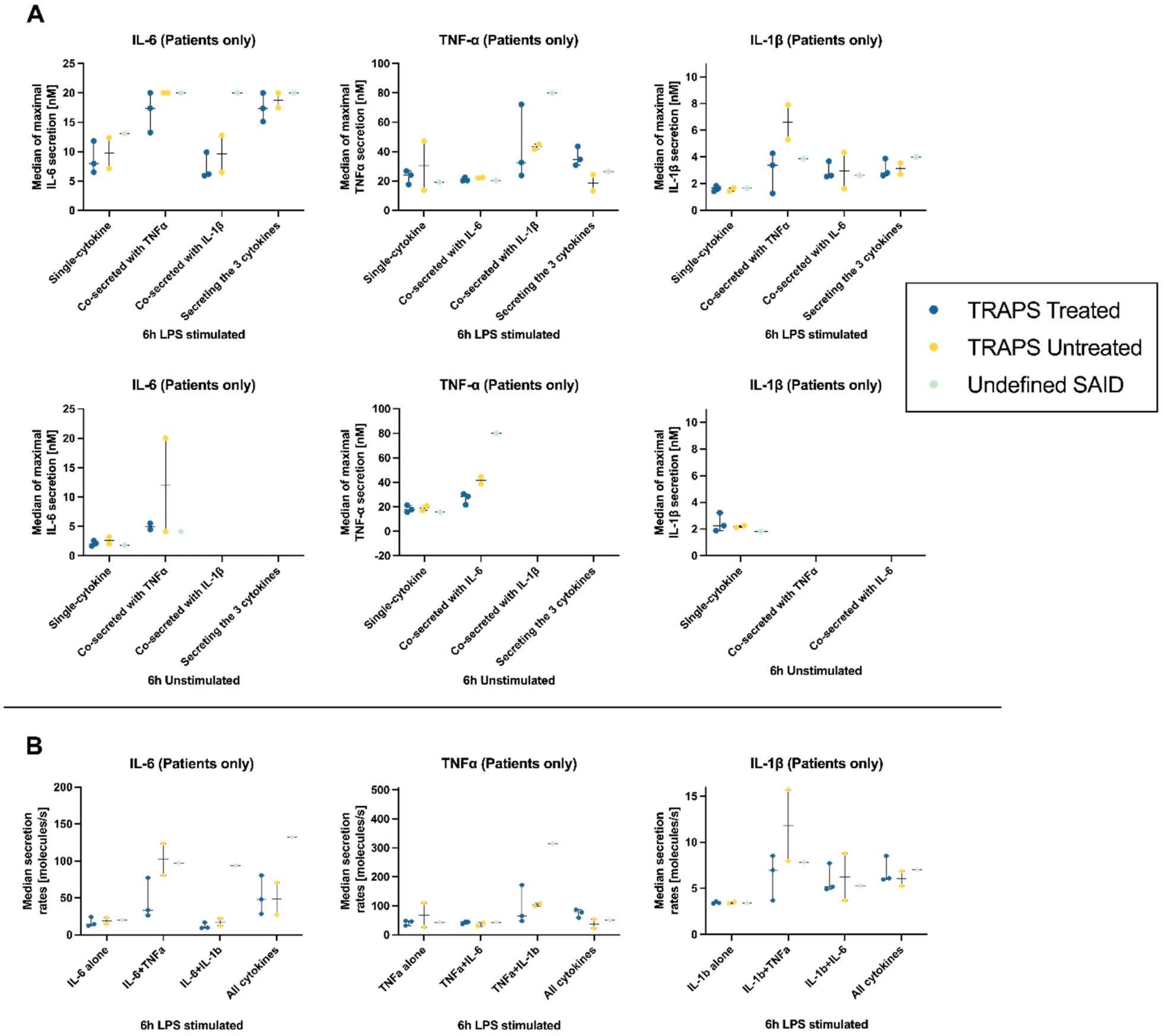

<u>Figure S3</u>: Median maximum concentration and secretion rates of the patients' samples to visualize the spread of the replicates, the median of the medians was used for the comparison to the healthy controls. The lines depict the median ± IQR 95%. The median was not extracted if less than 5 SCs were detected in the subgroup.

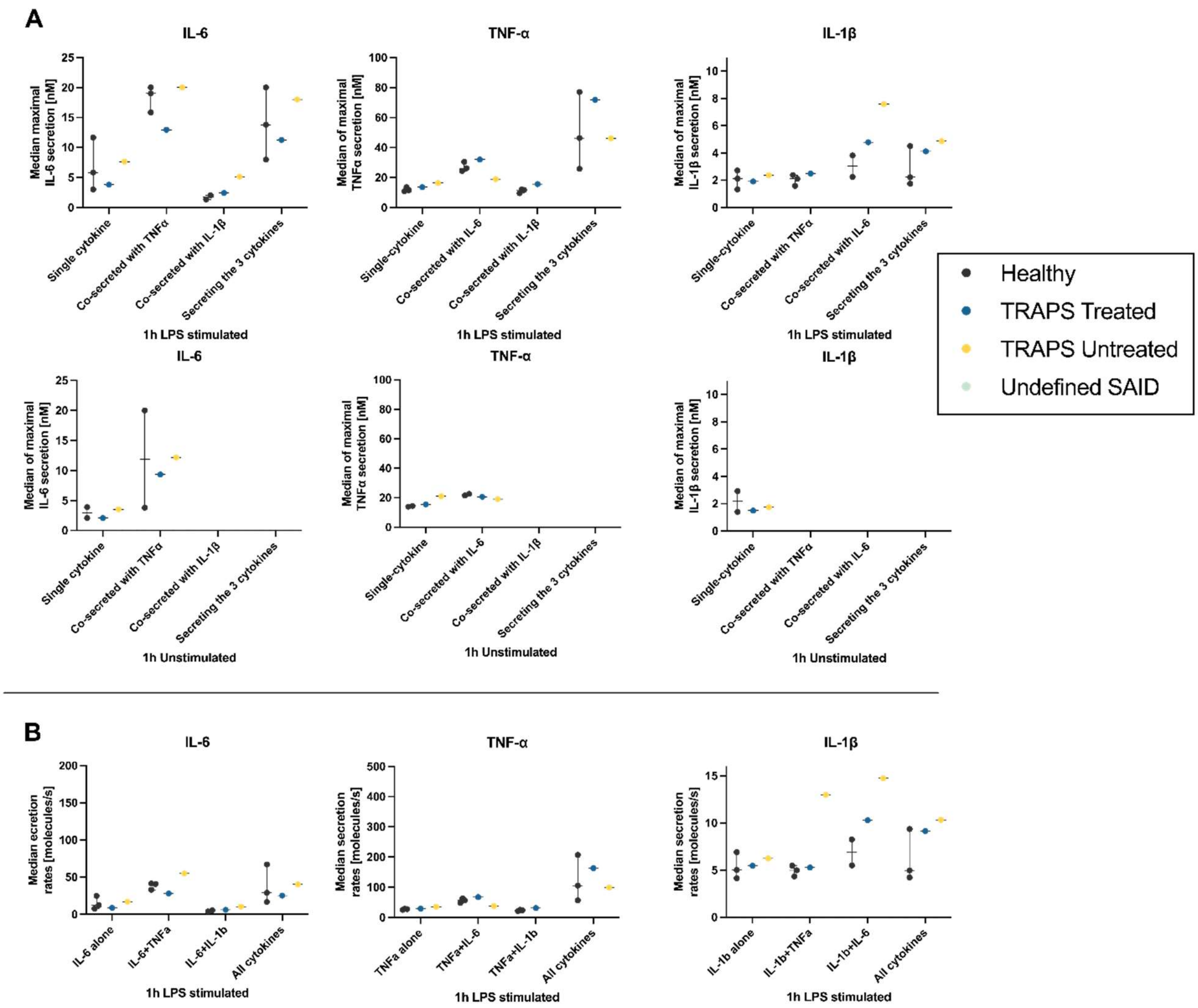

Figure S4: Median maximal secreted concentration (A) or secretion rate (B) of IL-6, TNF-α or IL-1β after 1 hour of stimulation with LPS or unstimulated. For each cytokine, each dot corresponds to the median of the maximum secreted concentration over the measurement time. The line depicts the median ± IQR 95%. Healthy control group N=3 (2 for unstimulated), n=1; TRAPS Treated group N=1, n=1; TRAPS Untreated group N=1, n=1. The median was not extracted if less than 5 SCs were detected in the subgroup.

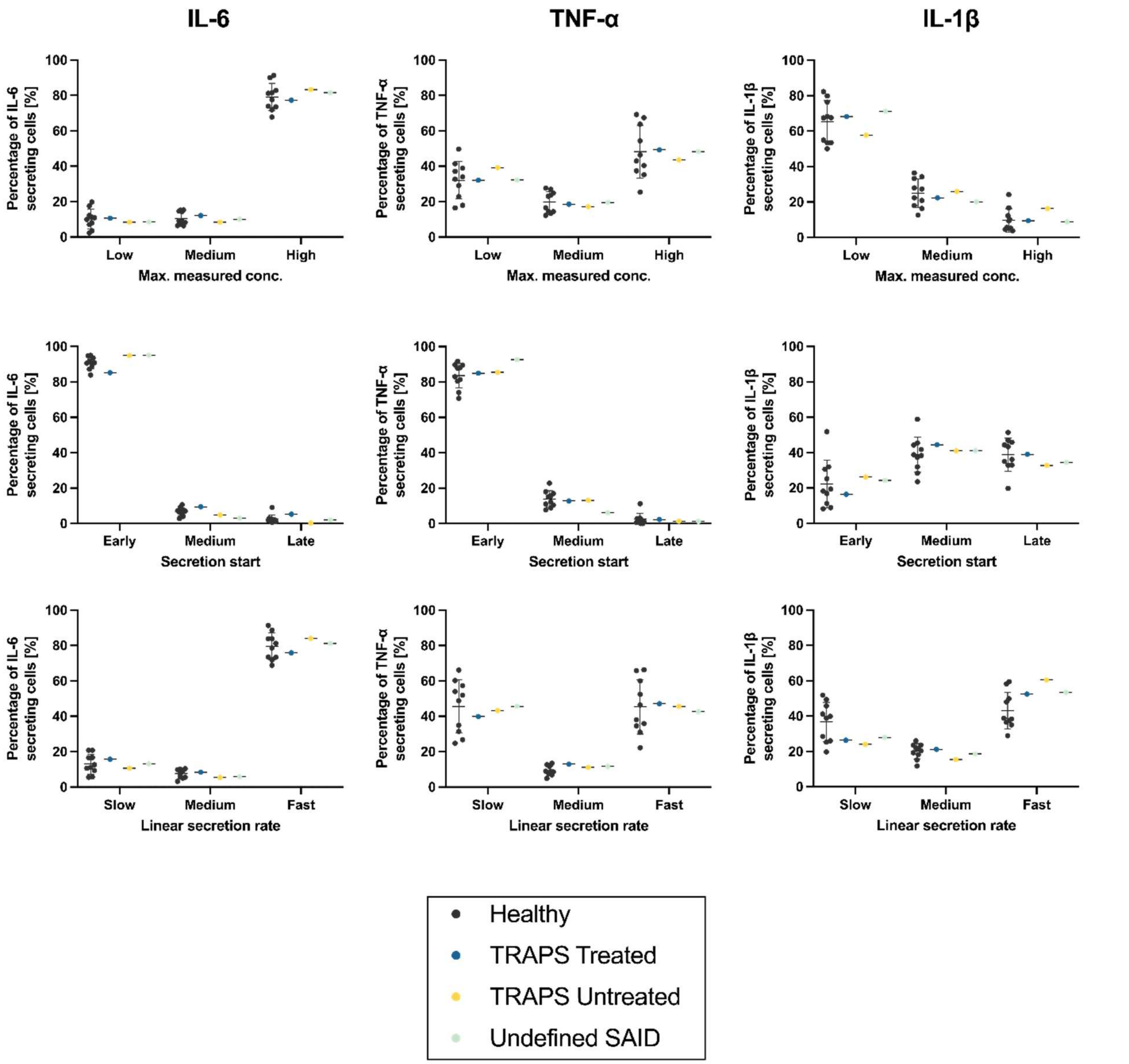

Figure S5: Detailed analysis of the secretion dynamic pattern of single cells secreting IL-6, TNF-α and IL-1β 6 hours following LPS stimulation. SCs were classified into different secretor types according to the following criteria: Maximum concentration, identifying high secretors with a maximal secretion higher than ¾ of the measurement range (>15 nM for IL-6, >60 nM for TNF-α, >9.375 nM for IL-1β) and low secretors with a maximal secretion lower than ¼ of the measurement range (<5 nM for IL-6, <20 nM for TNF-α, < 3.125 nM for IL-1β); Secretion start with early secretors starting within 30min and late secretors starting after 120 min (same for each cytokine); Secretion rate with fast secretors having a secretion rate reaching the maximum concentration in 30 min or less (>67 molecules/s for IL-6, >268 molecules/s for TNF-α, >42 molecules/s for IL-1β) and slow secretors having a secretion rate lasting longer than 60 min to reach max (<33 molecules/s for IL-6, <134 molecules/s for TNF-α, <21 molecules/s for IL-1β).

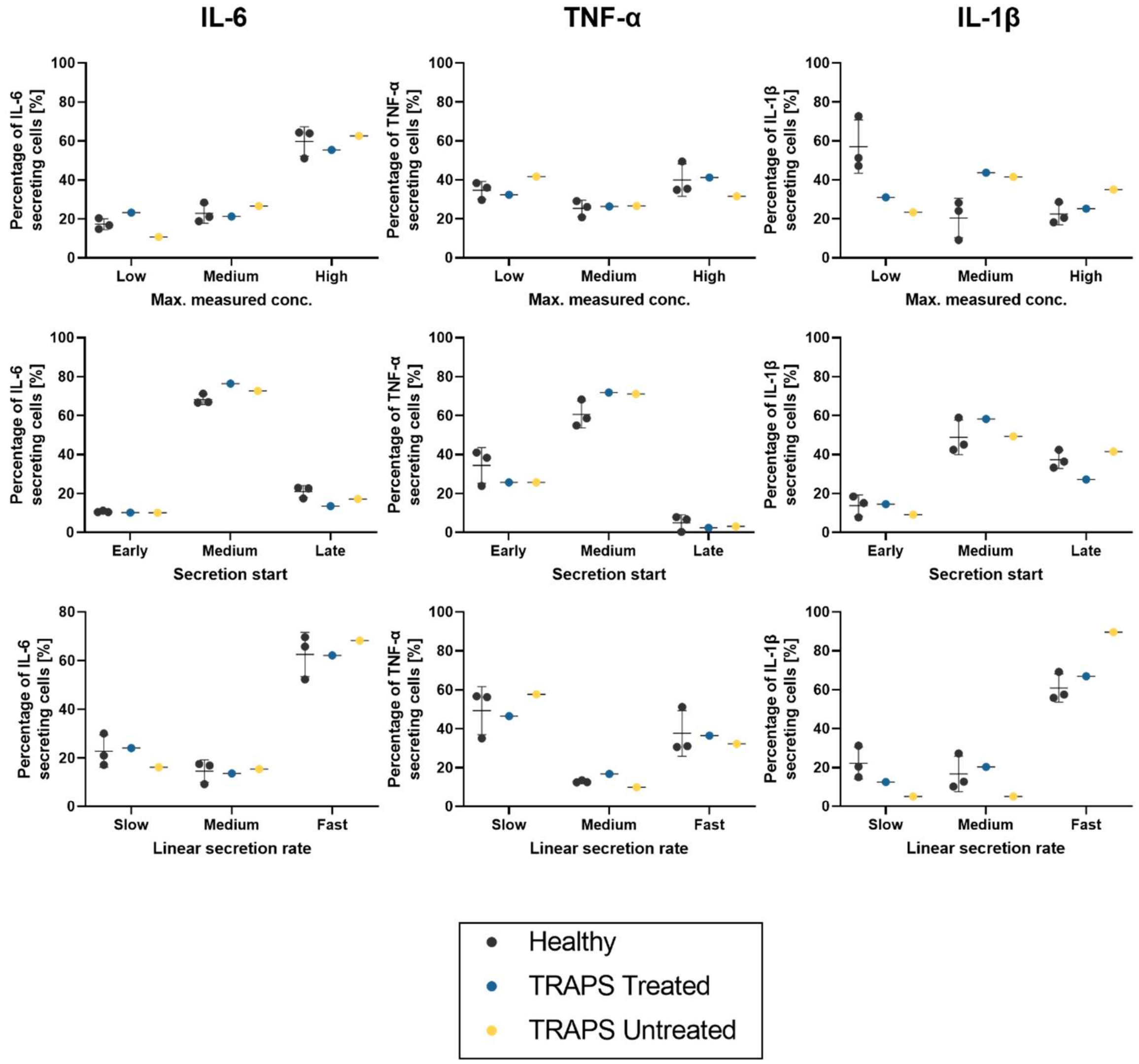

Figure S6: Detailed analysis of the secretion dynamic pattern of single cells secreting IL-6, TNF-α and IL-1β following 1-hour LPS stimulation. Secreting cells were classified into different secretor types according to the following criteria: Maximum concentration, identifying high secretors with a maximal secretion higher than ¾ of the measurement range (>15 nM for IL-6, >60 nM for TNF-α, >9.375 nM for IL-1β) and low secretors with a maximal secretion lower than ¼ of the measurement range (<5 nM for IL-6, <20 nM for TNF-α, < 3.125 nM for IL-1β); Secretion start with early secretors starting within 30min and late secretors starting after 120 min (same for each cytokine); Secretion rate with fast secretors having a secretion rate reaching the maximum concentration in 30min or less (>67 molecules/s for IL-6, >268 molecules/s for TNF-α, >42 molecules/s for IL-1β) and slow secretors having a secretion rate lasting longer than 60min to reach max (<33 molecules/s for IL-6, <134 molecules/s for TNF-α, <21 molecules/s for IL-1β).

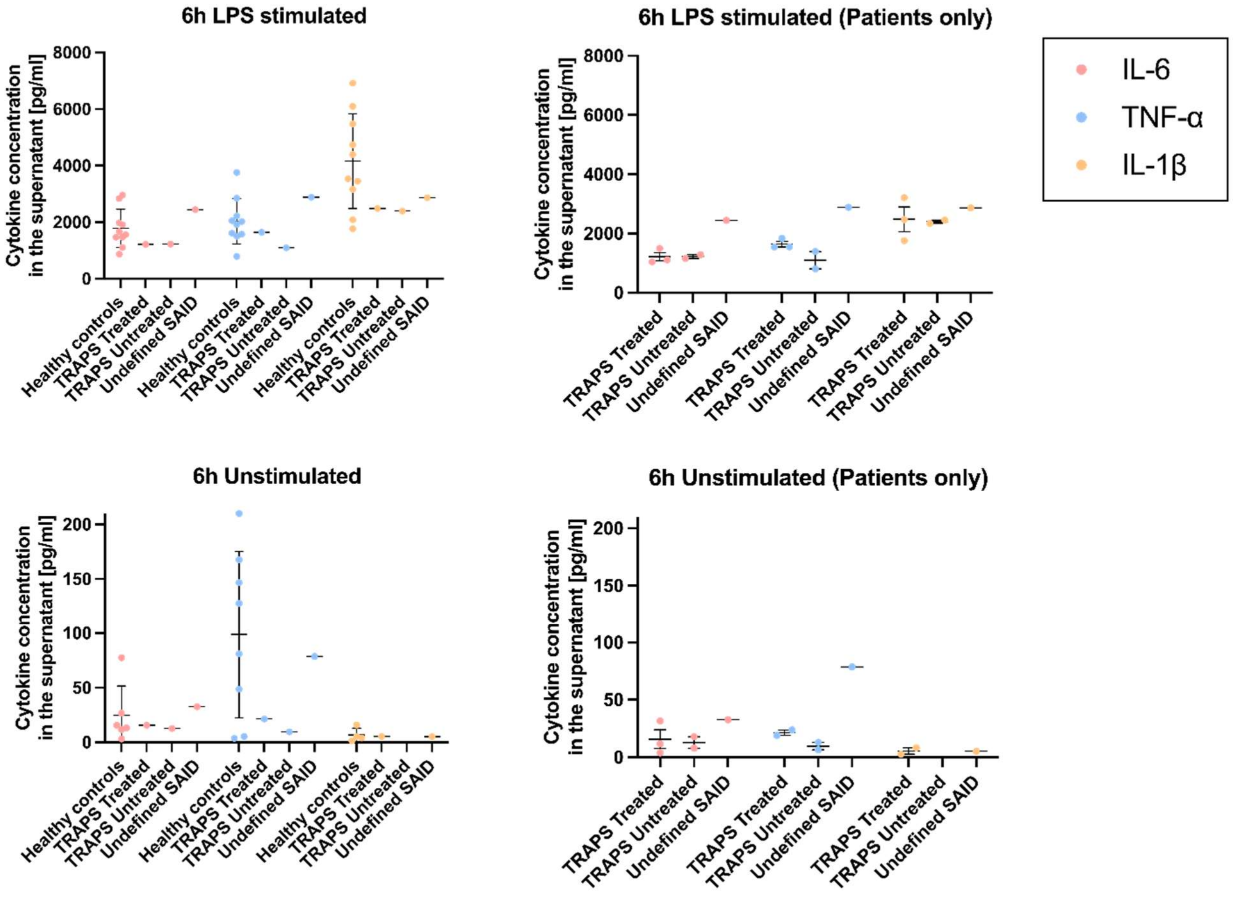

<u>Figure S7</u>: Cytokine concentrations in the supernatant of the cells before encapsulation. IL-6 (pink), TNF-α (blue), and IL-1β (yellow) were measured by ELISA in the supernatant of cells stimulated or not for 6 hours with LPS. Depicted with mean ± SD, the replicates of the patients' samples are shown with mean ± SEM. No significant differences between the groups were found using a One-way ANOVA for each cytokine. Following 6-hours LPS stimulation, healthy control group N=10, n=1; TRAPS Treated group N=1, n=3 (triplicate); TRAPS Untreated N=1, n=2 (duplicate); Undefined SAID N=1, n=1. The same number of samples were tested following 6 hours without stimulation, however, due to the sensibility of the ELISA assays, the sample number decreased according to the cytokine to N=6/8/4 (IL-6, TNF-α, IL-1β respectively) in the healthy control group; N=1, n=3/2/2 in Treated TRAPS; N=1, n=2/2/0 in TRAPS Untreated and N=1, n=1/1/1 in undefined SAID.
